# Auditory Cortex Represents Both Pitch Judgments and the Corresponding Acoustic Cues

**DOI:** 10.1016/j.cub.2013.03.003

**Published:** 2013-04-08

**Authors:** Jennifer K. Bizley, Kerry M.M. Walker, Fernando R. Nodal, Andrew J. King, Jan W.H. Schnupp

**Affiliations:** 1 Department of Physiology, Anatomy and Genetics, University of Oxford, Oxford OX1 3PT, UK; 2 Ear Institute, University College London, London WC1X 8EE, UK

## Abstract

The neural processing of sensory stimuli involves a transformation of physical stimulus parameters into perceptual features, and elucidating where and how this transformation occurs is one of the ultimate aims of sensory neurophysiology. Recent studies have shown that the firing of neurons in early sensory cortex can be modulated by multisensory interactions [1–5], motor behavior [1, 3, 6, 7], and reward feedback [1, 8, 9], but it remains unclear whether neural activity is more closely tied to perception, as indicated by behavioral choice, or to the physical properties of the stimulus. We investigated which of these properties are predominantly represented in auditory cortex by recording local field potentials (LFPs) and multiunit spiking activity in ferrets while they discriminated the pitch of artificial vowels. We found that auditory cortical activity is informative both about the fundamental frequency (F0) of a target sound and also about the pitch that the animals appear to perceive given their behavioral responses. Surprisingly, although the stimulus F0 was well represented at the onset of the target sound, neural activity throughout auditory cortex frequently predicted the reported pitch better than the target F0.

## Highlights

► Auditory cortical responses were recorded while ferrets discriminated pitch shifts ► LFP and multiunit activity are sensitive to the sound’s fundamental frequency (F0) ► Neural activity related to animals’ reported pitch increases throughout the trial ► Cortical responses were more informative about behavioral choices than the sound F0

## Results and Discussion

We recorded neural activity in trained ferrets while they performed a two-alternative forced-choice discrimination task and investigated whether single-trial neural activity better predicted the physical properties of the sound that had been presented to the animal or the perceived sound qualities that the animal reported via its behavioral choice. We recorded LFPs as well as spiking activity. The LFP reflects the spatially weighted average of the synaptic transmembrane currents [10, 11], representing both the inputs to a brain area and the local processing that occurs there and providing a unique insight into cortical network activity [12]. The temporal structure of the LFP reflects bottom-up sensory information, which can be modulated by cognitive processes [13–15]. Therefore, the LFP can provide additional information to that provided by single-neuron activity.

How the cues that underlie pitch perception are represented in the cortex remains controversial [16, 17]. Although there is evidence for a specialized pitch center [18, 19], a more broadly distributed network of “pitch sensitivity” has been reported in both humans [20–22] and animals [23–25]. Demonstrating whether neural activity within and beyond the putative pitch center correlates with reported pitch may help resolve the question of which representations of stimulus periodicity contribute to perception.

Trained ferrets indicated whether the fundamental frequency (F0) of a “target” sound was higher or lower than a preceding “reference” sound by licking one of two spouts for water reward [26]. The reference F0 was held constant in each behavioral testing session, whereas the F0 of the target sound varied randomly from trial to trial across a two-octave range. Multielectrode arrays were implanted into auditory cortex (see Figure S1 available online). Neural activity was recorded during the animal’s twice-daily testing sessions for one year, during which time the electrodes were systematically advanced in depth, approximately weekly.

Trials were categorized according to whether the F0 of the target sound was higher or lower than that of the reference or whether the animal made a left (“lower pitch”) or right (“higher pitch”) choice. To explore the temporal relationships between acoustic stimuli, neural signals, and behavioral responses, we calculated the root mean square (RMS) amplitude of the LFP signal over 200 ms wide sliding temporal windows beginning at the onset of the target sound, with subsequent windows at 100 ms intervals. We also included a 200 ms window that started at the reference sound onset. Receiver operating characteristic (ROC) analysis [27] was used to assess how well the recorded neural signals encoded either the sound F0 or reported pitch. ROC analysis provides a criterion-free method for estimating the discriminability between two trial types (e.g., higher or lower pitch), given the observed distributions of the recorded LFP power. ROC analysis has been used successfully to explore the relationship between neural activity and behavioral responses in the somatosensory [28], visual [29], and, more recently, auditory systems [6, 30]. The area under the ROC curve (aROC) is mathematically equivalent to the performance of an ideal observer performing a two-alternative forced-choice task based on the neural signal [27], with a value of 0.5 reflecting chance-level performance and values of either 0 or 1 indicating perfect discriminability. We computed aROCs to quantify how well the LFP power predicted both the F0 of the stimulus (higher versus lower; termed aROC_F0_) and the response of the animal (right spout for higher, left for lower; termed aROC_choice_). Whether values above or below 0.5 are obtained depends on the arbitrary mapping of LFP amplitudes onto the two response classes (i.e., whether higher F0 values are associated with larger or smaller LFP amplitudes). We therefore constrained all aROC values to lie between 0.5 and 1. A bootstrapping procedure (see Experimental Procedures) was used to determine the statistical significance of aROC values.

**Figure 1:**
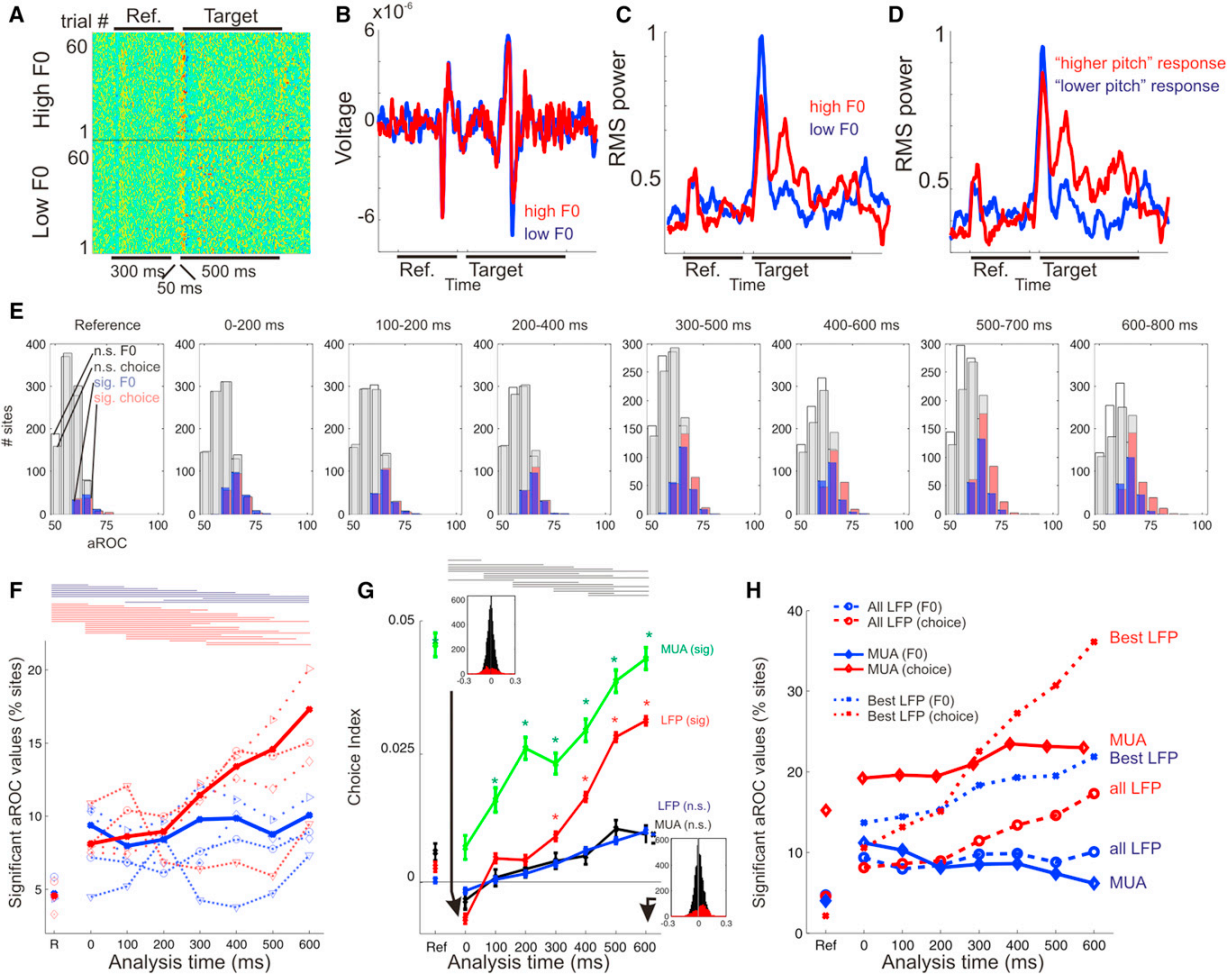
Cortical Activity Represents Both the Fundamental Frequency of a Stimulus and Its Reported Pitch (A) Local field potential traces. Each row depicts the voltage value on a single trial, with trials grouped according to whether the target F0 was higher or lower than that of the reference sound. The duration of the reference and target are depicted by the black bars above and below the plot. (B) Mean LFP from (A), averaged according to trial type (higher versus lower target F0). (C and D) RMS power (from A), calculated in 5 ms bins, smoothed with a ten-point moving average, and normalized. Trials are grouped according to either the target F0 (C) or the response made by the animal (D, left response corresponding to “lower pitch,” right response corresponding to “higher pitch”). (E) Histograms showing the best aROC_F0_ and aROC_choice_ values across recording sites for each analysis time window. Each panel shows the distribution of RMS aROC values calculated over a 200 ms window beginning either at reference-sound onset or at the time intervals shown, where 0 ms corresponds to the onset of the target sound and 500 ms corresponds to target-sound offset. The aROC values significantly greater than chance are plotted in blue (aROC_F0_) and red (aROC_choice_), whereas insignificant values are plotted in gray (aROC_F0_) and white (aROC_choice_). (F) The percentage of significant aROC_F0_ (heavy blue) and aROC_choice_ (heavy red) values for all (rather than just the best at each site, see Supplemental Experimental Procedures) recordings. Data from four individual animals are overlaid (thin lines).Horizontal bars indicate significant pairwise post hoc comparisons between time windows. (G) Choice index values. Sites where either the aROC_F0_ or aROC_choice_ was significant are plotted in red (LFP) or green (MUA), with an asterisk indicating values that are significantly different from zero (t test, p < 0.05/8). Nonsignificant sites are shown in blue (LFP) and black (MUA), with positive values indicating that aROC_choice_ > aROC_F0_. Horizontal bars indicate significant post hoc pairwise comparisons for the significant ROC values in the LFP data. (H) Comparison of MUA, best LFP, and all LFP data showing the data from all animals.

Figure 1 shows the LFP recordings on one electrode from a single behavioral session (top row; Figures 1A–1D). Figure 1E shows the distributions of aROC_F0_ and aROC_choice_ values obtained from four ferrets, reporting the best aROC value obtained at each recording site, and Figure 1F plots the proportion of sites with significant aROC values across all LFP recordings. The proportion of significant sites varied across analysis time windows (Figure 1F) (Kruskal-Wallis test, p < 1×10^−7^), and more sites showed significant choice-related activity (red line) than significant stimulus-related activity (blue line). We compared the stimulus-related versus response-related activity at each site directly using a choice index (CI), calculated as:

$$\text{CI}=\frac{\left({\text{aROC}}_{\text{choice}}-{\text{aROC}}_{\text{F0}}\right)}{\left({\text{aROC}}_{\text{choice}}+{\text{aROC}}_{\text{F0}}\right)}.$$

The resulting CI values were positive if the neural activity was more informative about the animal’s choice than the target F0 and negative if the opposite was the case (Figure 1G). If we consider only sites in which at least one of the aROC values was significant (Figure 1G, red line), the average CI is initially negative, indicating F0 dominance. However, CI values become increasingly positive throughout the duration of the trial, indicating that information about the animal’s sensory decision, rather than the target-sound acoustics, increasingly dominates the LFP signal. When we repeated these calculations for a data set of simultaneously recorded multiunit spiking activity (MUA, 1,140 recordings from 350 individual sites), we found an even greater sensitivity to behavioral choice (Figure 1G, green line). The MUA and LFP data therefore demonstrate similar trends, though more MUA recordings exhibited choice sensitivity than LFP recordings (Figure 1H). Additional analyses, which calculated choice probabilities in a stimulus-independent manner [31], further confirmed that the neural responses reflect the animals’ sensory decision (Figure S2).

**Figure 2:**
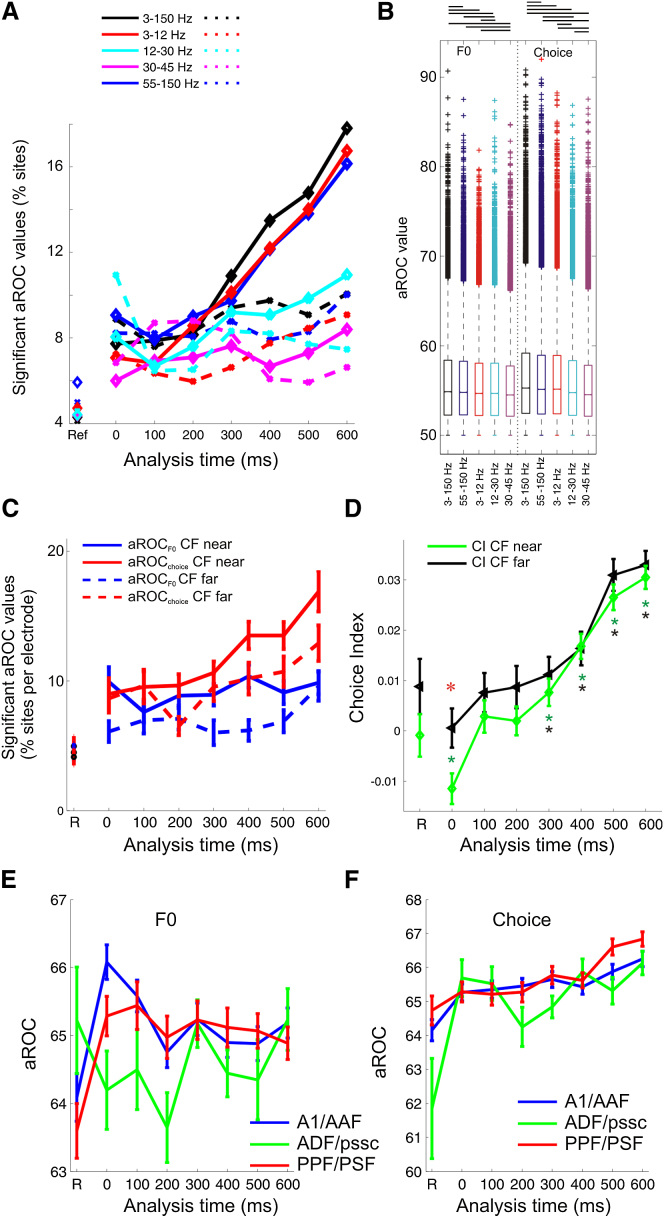
Dependence of Choice and Periodicity Sensitivity on LFP Spectral Band and Recording Location (A) Analysis of LFP by spectral band. The LFP was filtered into the bands indicated, and the percentage of significant aROC_F0_ (stippled lines) or aROC_choice_ (solid lines) values is shown in different analysis time windows. (B) aROC values obtained with significant differences indicated by horizontal bars. Frequency bands are ranked left to right from most to least informative. (C) Electrodes were grouped into those with near and far CFs relative to the stimulus F0. The percentage of significant sites (mean ± SEM) across electrodes is shown in different time windows. (D) Choice index values across all recording sites which had a significant aROC_F0_ or aROC_choice_ value, grouped by CF (mean ± SEM). Asterisks indicate which time points were significantly different from zero (t test, p < 0.05 / 8 = < 0.0063). Red asterisk marks the only time point at which the distribution of CI values was significantly different (two sample t test, p = 0.011). (E and F) aROC values obtained for significantly informative recording sites according to cortical field.

Previous studies have demonstrated that sensory information is not distributed equally across different LFP bands [32, 33]. Therefore, we filtered our LFP signals to examine the neural responses in four frequency bands: <12 Hz, 12–30 Hz (beta), 30–45 Hz (low gamma), 55–150 Hz (high gamma, thought to correspond most closely to MUA and BOLD signals [34]). A comparison of the normalized power spectra between active and passive conditions (Figure S2) revealed that the LFP power increased in frequencies > 12 Hz during the task. Both the proportion of significant aROC values (Figure 2A) and the magnitude of aROC values obtained (Figure 2B) suggested that the broadband signal (3–150 Hz; black line) was most informative about both F0 and pitch judgment. Consistent with previous studies [32], when considering restricted frequency bands, the high gamma (blue line) and <12 Hz (red line) frequency ranges were most informative than the other 2 bands (Figure 2A and 2B).

We next investigated whether the characteristic frequency (CF) of a recording site influenced the likelihood of the LFP signal discriminating F0 or behavioral choice. “Near-CF” sites were those with a CF within an octave of the highest or lowest F0 target for the relevant testing session, and “far-CF” sites were those with CFs outside of this range. Near-CF sites were more likely to have informative aROC_F0_ values (Kruskal-Wallis test; p = 7 × 10^−7^) and aROC_choice_ values (p = 0.0003) (Figure 2C). Both near- and far-CF sites had predominantly positive choice index values, with near-CF sites more likely to show stimulus dominated CIs at target-sound onset (Figure 2D).

Although neural sensitivity to sound F0 is broadly distributed in the auditory cortex of anesthetized ferrets [24, 25] and awake macaques [23], studies in passively listening humans [19, 35–37] and marmosets [18] suggest that discrete areas of auditory cortex might be specialized for pitch processing. We therefore divided recordings into those made in three regions: the primary (A1 and AAF) fields; the posterior fields (PSF and PPF); and the anterior dorsal field (ADF) and pseudosylvian sulcal cortex (pssc) [38]. We found no significant differences across these regions in the proportion of recording sites that had significant aROC_FO_ or aROC_choice_ values (Figures S3A and S3B). Nevertheless, the aROC_F0_ values were significantly higher in the primary fields than the anterior fields (Figure 2E) (Kruskal-Wallis test, p = 0.035, post hoc comparisons A1/AAF > ADF/pssc, p < 0.05), and aROC_choice_ values were significantly higher in the posterior fields than in other regions (Kruskal-Wallis test, p = 0.0006, post hoc comparisons A1/AAF < PSF/PPF, p < 0.05). Although differences across cortical fields are modest, our data suggest that stimulus F0 is better represented in primary cortex, and that stronger choice-related activity emerges in higher fields [39].

**Figure 3:**
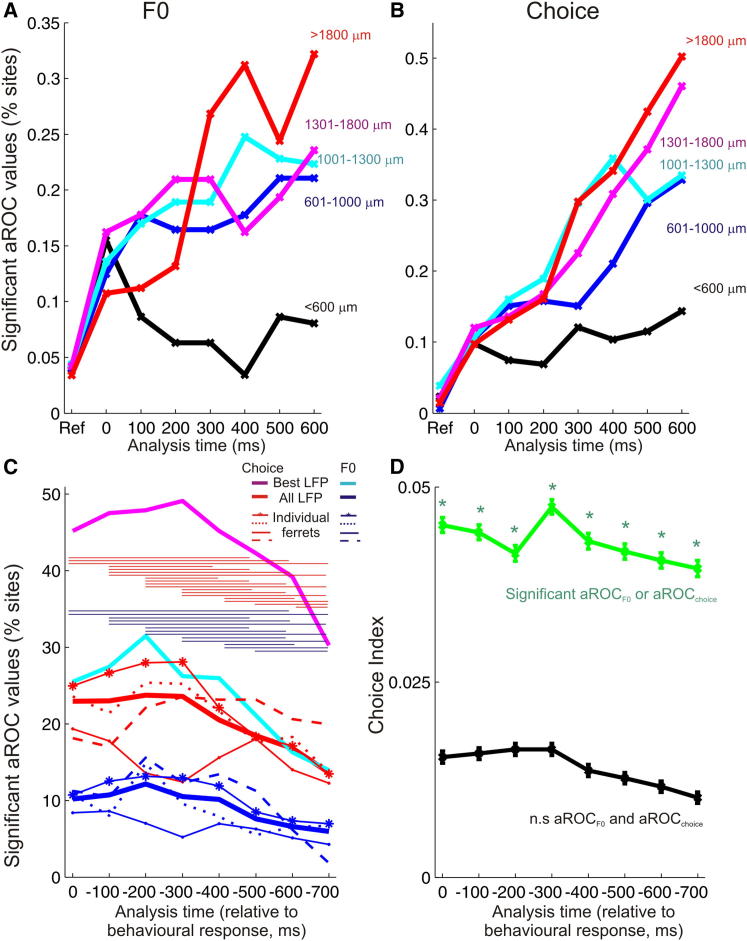
Dependence of Choice and Periodicity Sensitivity on Cortical Depth and Response Time (A and B) Percentage of recording sites with significant aROC_F0_ (A) and aROC_choice_ (B) in different analysis time windows, arranged according to recording depth. (C) Percentage of significant sites when responses are analyzed at different time points prior to the time of the animal’s response (0 ms). Data are shown for the best sites (cyan, aROC_F0_; purple, aROC_choice_), the mean across all sites (blue, aROC_F0_; red, aROC_choice_), and for individual animals (thin lines). Significant differences across time for aROC_F0,_ (blue) and aROC_choice_ (red) are shown with horizontal bars. (D) Choice index values for response timed analysis, across all recording sites (mean ± SEM).

Neural sensitivity to both F0 and reported pitch varied according to cortical depth (Figures 3A and 3B), with a greater percentage of deep recording sites having significant aROC_F0_ and aROC_choice_ values than those sites recorded in the superficial layers. An important exception to this is the earliest time bin, in which aROC_F0_ and aROC_choice_ values were equally likely to be significant across all layers.

Finally, we examined neural responses relative to the animals’ behavioral response time rather than the stimulus onset. LFP RMS power values were computed in 200 ms windows running backward from the time the animal triggered the response spout. The proportion of sites with significant aROC_choice_ values increased when analyzed this way. Choice values were highest in the time window that spanned 500–300 ms before the animal made its response (Figure 3C). Choice index values were highly positive throughout the response-timed analysis, and as with the stimulus-timed analysis, recordings in deeper layers were more likely to be significant than those in superficial layers (Figure 3D; Figures S3D and S3E).

Our data therefore show that activity in early auditory cortex, including the primary areas, carries information about both the sound F0 and the animal’s impending sensory decision. What does this choice-related activity represent? Sensory evidence accumulated over time is thought to be combined with factors such as the prior probability of a particular stimulus occurring and reward expectation to form a decision variable that guides behavior [40]. Our task was not designed to tease apart the extent to which neural activity represents sensory evidence, perception itself, or decision making. However, given that neural activity is an imperfect predictor of both F0 and choice, it seems unlikely that the activity we observe represents the decision variable itself. Rather, because the prestimulus activity can be predictive of the animals’ behavioral judgment (Figure 1G), what we observe may result from correlations in the activity structure of the neurons that contribute to the perceptual decision [41, 42].

The buildup of activity related to the reported F0 throughout the trial is consistent with feedback from higher areas reflecting either decision or motor-related activity [7, 42, 43]. This buildup is consistent with previous work showing that single neurons do not have significant choice probabilities when short analysis windows focused over stimulus onset are used [6]. Indeed, neural signatures of category discrimination are present over several seconds [44], and the responses of auditory cortical neurons can remain informative about the stimulus for up to 500 ms after sound offset [44, 45].

The few previous studies which have investigated how auditory cortical activity relates to an animal’s behavioral choice have generally investigated sound detection tasks ([6, 30], though see [46]). Our behavioral task required that on each trial the ferret make a pitch-discrimination judgment. Consequently, the attentional demands and the animals’ expectations of reward were the same on each trial and, importantly, were not contingent on the class of stimulus presented. Although attention and reward expectation undoubtedly shape neural activity in auditory cortex [6, 47–49], such influences should not have preferentially affected one trial type more than another. Importantly, we found increases in LFP amplitude were equally likely to predict either “higher” or “lower” responses (Figure S2A). Although some studies have observed decision-related activity in auditory cortex [6, 50], others have not [30, 46]. It seems likely that differences in task design and the level of abstraction required by animals performing these tasks might be one factor that accounts for these differences.

When we considered how F0 and choice-related activity varied across different parts of auditory cortex, we observed only modest differences. This may indicate that the neural basis of pitch perception is distributed across multiple fields. However, recording sites with CFs near the target frequency range showed greater F0 sensitivity and greater choice-related activity. Since these near-CF sites all had BFs < 3.2 kHz, the most informative sites were likely to be on the low-frequency borders of the primary and nonprimary fields. Although this observation is consistent with the idea of a specialized pitch-processing area [38, 51], pitch-related activity can be observed throughout auditory cortex [22, 24, 25]. Demonstrating which signals play a causal role in pitch perception will require that future studies manipulate neural firing during discrimination tasks.

## Experimental Procedures

### Animals

A total of four adult, female, pigmented ferrets (*Mustela putorius*) were used in this study. All experiments were approved by the local ethical review committee and carried out under license from the UK Home Office in accordance with the Animal (Scientific Procedures) Act (1986). Ferrets were housed in groups of two or three, with free access to food pellets and water bottles. On the day before behavioral testing, water bottles were removed from the home cage. Testing runs lasted for ≤ 5 days, with at least 2 days between each run. On testing days, ferrets received drinking water as positive reinforcement. Regular otoscopic and tympanometric examinations were carried out to ensure that the animals’ ears were clean and healthy.

### Acoustic Stimuli

Artificial vowel sounds were composed of click trains that were band-pass filtered to add “formants” centered at 430 Hz, 2,132 Hz, 3,070 Hz, and 4,100 Hz and were then enveloped with 5 ms rise and fall times. These formants correspond to the first four formants of the English vowel /*i*/ (as in “pill”). The repetition rate of the click train from which the vowel was generated determined the periodicity (fundamental frequency, F0) and therefore the perceived pitch.

### Behavioral Testing

Ferrets were trained to report the direction of an F0 change [26]. On each trial, animals were presented with two sound bursts: a 200 ms reference sound, followed by a 500 ms target sound. Animals were trained to respond to a spout to their left if the target F0 was lower than the reference F0 and to the right if the target F0 was higher than the reference. Our previous studies indicated that the ferrets were performing a periodicity-pitch task, rather than using other covarying acoustical cues, such as spectral density. They showed that ferrets generalized the lower/higher task to pure tone stimuli [26], and disrupting the temporal regularity of the click trains, which decreases pitch salience while maintaining a constant spectral density, reduced the ferrets’ ability to perform this task [24]. During training and initial testing, as many as 20 target sounds were randomly presented across a range of ±1 octave from the reference sound. However, once we began neural recording sessions (6 months to 2 years after training had commenced), we decreased the number of targets to seven evenly and logarithmically spaced F0s around the reference F0 (± ≤1 octave), including catch trials where the target F0 = reference F0.

### Data Analysis

#### Local Field Potentials

Broadband voltage signals, sampled at 25 kHz, were band-pass filtered between 5 and 300 Hz and then downsampled to 1 kHz. Correlated 50 Hz activity across channels (due to mains electricity noise) was removed using the NoiseTools MATLAB toolbox [52, 53]. Individual channel 50 Hz noise was further removed by transforming signals into the Fourier domain, attenuating the peak of 50 Hz by smoothing from 48 to 52 Hz, and then inverse-Fourier transforming the data back into the time domain. Additional data processing details are available in the Supplemental Experimental Procedures.

#### Analysis Time Windows

Trials were initiated when the animal poked its nose in the central start spout, which, after a variable delay (400–1,000 ms), triggered the presentation of the reference sound. We recorded neural data from 200 ms (three animals) or 400 ms (one animal) before the onset of the reference sound, until 400 ms after the animal’s response had been registered at one of the lateral response spouts. The average duration of a trial was 1,665 ± 103 ms from reference-sound onset (mean ± SD). We therefore did not extend our analysis time beyond 900 ms post reference onset because the solenoid system that delivered the water reward generated an audible click that often elicited an evoked response on the electrode.

## Supplemental Information

Document S1. Figures S1–S3 and Supplemental Experimental Procedures
