# Supplementary material for: Auditory Cortex Represents Both Pitch Judgments and the Corresponding Acoustic Cues
Source: Curr Biol. 2013 Apr 8;23(7):620–5. doi: 10.1016/j.cub.2013.03.003 (PMC3696731; doi:10.1016/j.cub.2013.03.003)
Supplement: Document S1. Figures S1–S3 and Supplemental Experimental Procedures [file mmc1.pdf]

Current Biology, Volume *23*

Supplemental Information

Auditory Cortex Represents Both Pitch Judgments and the Corresponding Acoustic Cues

Jennifer K. Bizley, Kerry M.M. Walker, Fernando R. Nodal, Andrew J. King, and Jan W.H. Schnupp

Supplemental Inventory

- Supplemental Figures:
  - S1, Related to Figure 1. *Figure S1 provides methodological details to assist in interpreting the data presented within the manuscript.*
  - S2, Related to Figure 2. *Figure S2 contains stimulus independent choice probability calculations and details of the change in LFP spectral composition observed relating to the data presented in figure 2.*
  - S3, Related to Figure 3. *Figure S3 contains additional single-animal measures of the difference in F0/choice sensitivity by cortical depth, and response-timed measures by cortical depth to complement response-timed measures in Figure 3.*
- Supplemental Experimental Procedures
  - Surgical protocol
  - Data pre-processing procedures
  - Data analysis methods
- Supplemental References

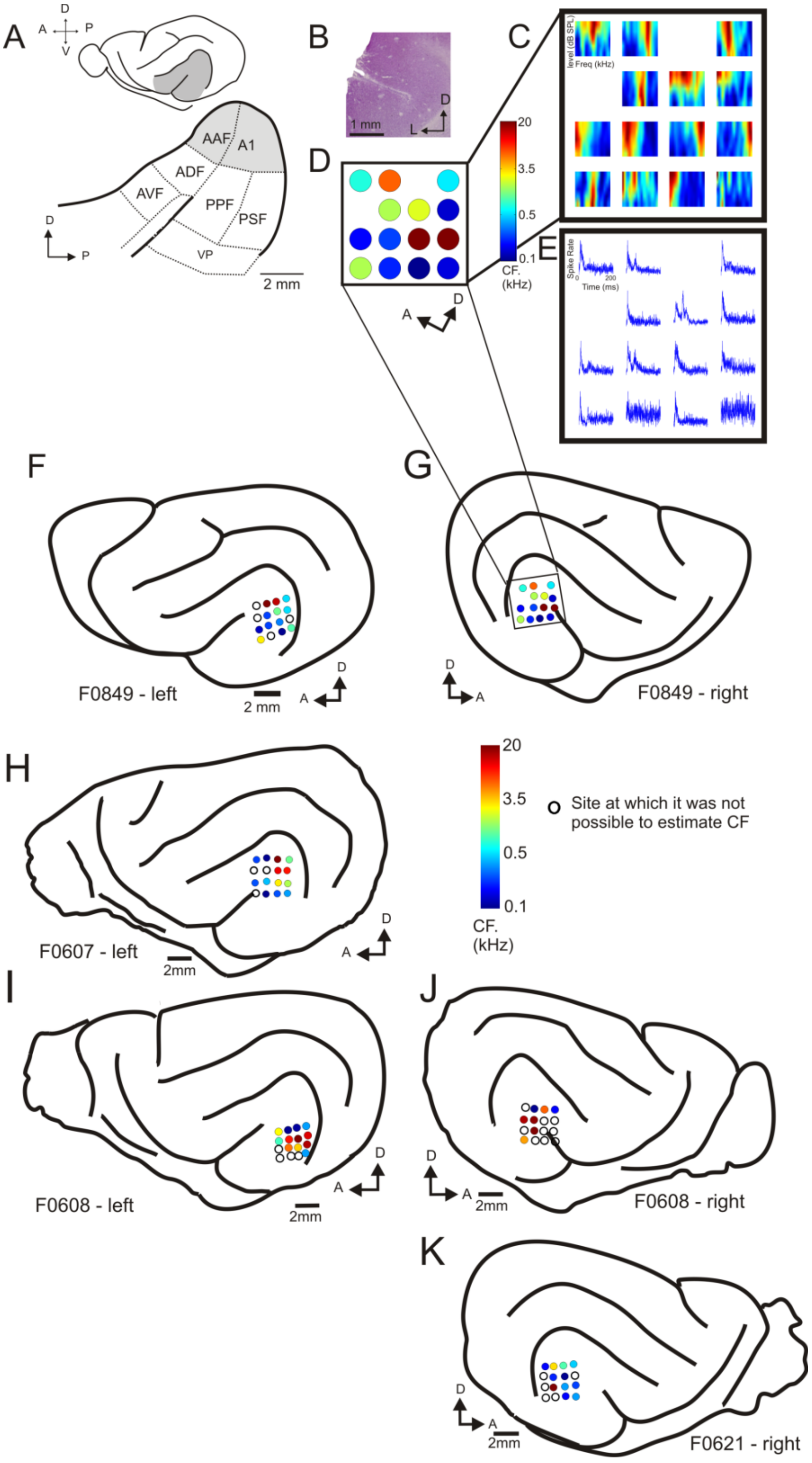

Figure S1, Related to Figure 1 and Experimental Procedures. Defining Recording Locations
(A) The location of ferret auditory cortex in the left hemisphere of F0849. The ectosylvian gyrus is shaded in gray in the picture of the whole brain, and enlarged below with the location of each auditory

field labeled. A1; primary auditory cortex, AAF; anterior auditory field, ADF; anterior dorsal field, AVF; anterior ventral field, PPF; posterior pseudosylvian field, PSF; posterior suprasylvian field, VP; ventral posterior field; A, anterior; D, dorsal; P, posterior; V, ventral.

(B) Cresyl violet stained section showing the location of a recording tract.

(C) The averaged frequency response area for each recording electrode is shown for all 14 of the electrodes that we were able to advance into the brain in this animal.

(D) Schematic showing the characteristc frequency measured for each electrode. Color scale as per G, below. White circles indicate sites at which it was not possible to derive a CF value; circles omitted from the 4 x 4 grid indicate sites at which we were unable to advance the electrode into the brain.

(E) Tone evoked multiunit PSTHs averaged across recording depths, frequencies and intensities for the electrodes shown in *C* and *D.*

(F) Drawing of F0849's left brain (as in A), with the location of the recording electrodes depicted and colored according to their characteristic frequency. Electrodes in which we were able to record neural activity, but not able to estimate a characteristic frequency, are shown as white circles.

(G) The location of recording sites in right auditory cortex for ferret 0849 (as in C-E).

(H) The location of recording sites in left auditory cortex for ferret 0607 (no recordings were made in the right auditory cortex of this animal).

(I and J) The location of recording sites in right and left auditory cortex for ferret 0608.

(K) The location of recording sites in right auditory cortex of ferret 0621. In both this case, and ferret 0607, electrode arrays were implanted bilaterally, but the arrays implanted on one side never yielded neural signals.

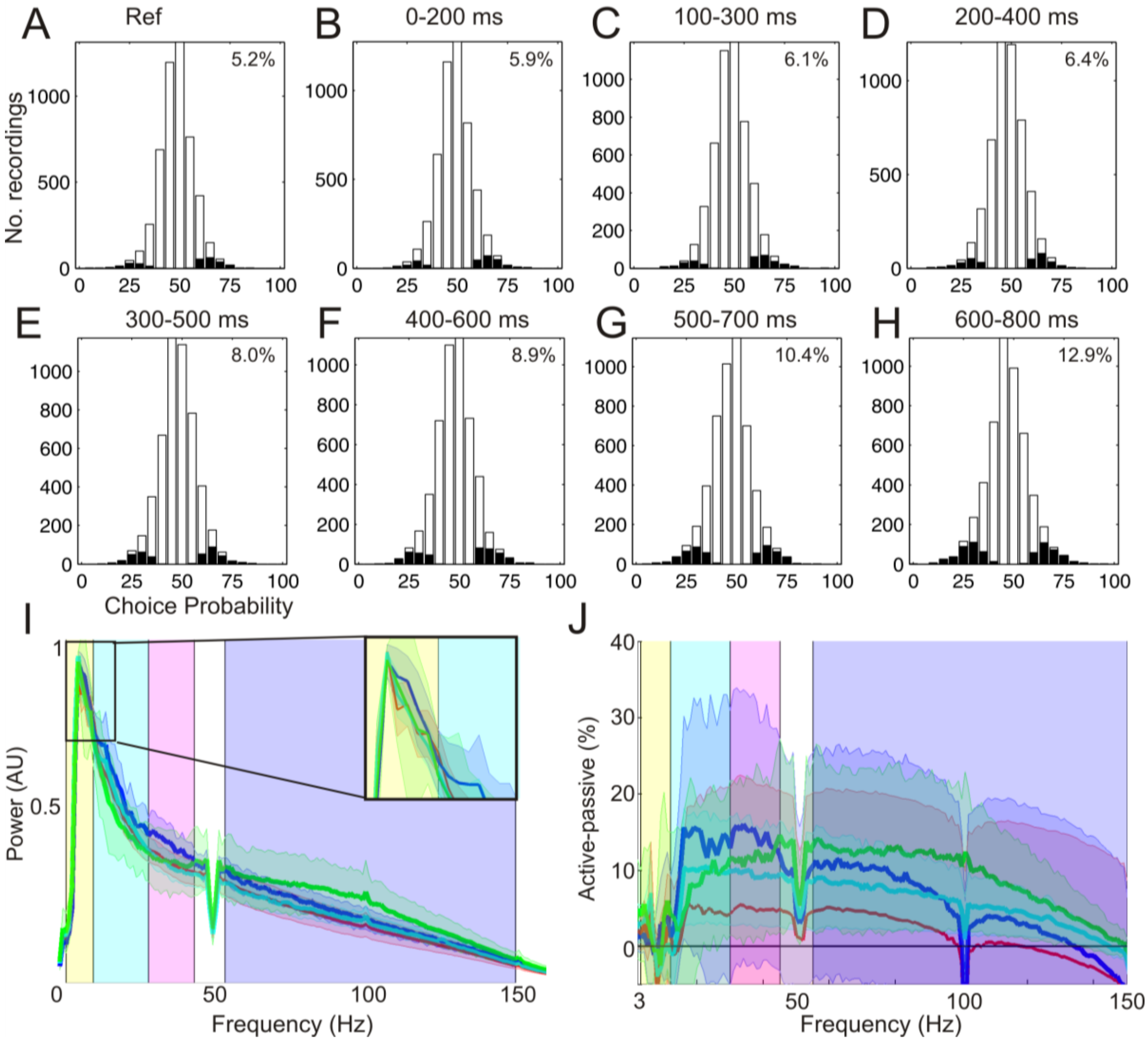

Figure S2, Related to Figures 1 and 2. Stimulus-Independent Calculation of Choice Probability
While our study design included catch trials we were unable to compute meaningful choice probabilities from them as they totaled too few in number. We therefore adopted a different approach based upon the methods of Newsome and colleagues [1, 2] which allowed us to remove the confound of differential neural sensitivity to the stimulus from measures of choice sensitivity. For each stimulus (i.e. target pitch) the LFP RMS power values were first normalized according to their mean and standard deviation. This method is sometimes referred to as the 'grand choice probability'. The z-scored values were then collapsed across different stimulus types (with the caveat that to be included there be at least three 'lower' and three 'higher' responses for that target pitch). Choice probability (CP) values were then computed using ROC analysis. Significance was assessed with a 2000 iteration permutation test (i.e. bootstrapping). We performed this analysis on our recordings. Broadly speaking we found the results to be in good agreement with our $aROC_{choice}$ results; i.e. choice probabilities increased throughout the trial. Overall, slightly fewer sites had a significant CP as calculated this way (peaking 13% as opposed to 18% of recordings). The histograms in Figure S2A-H show the distributions of all measured CPs, at each

analysis time window, with those that were significantly different from chance colored black. We decided not to constrain this analysis such that values were always >0.5 (by flipping the contingency between high power and response type). This figure therefore illustrates an additional point: that recording sites signaling ‘lower pitch’ with an increase in power were as common as those signaling it with a decrease in power.

(A–H) Stimulus-independent choice probability measures. The distribution of computed choice probability values is plotted for each analysis time window.

(I) Distribution of power across frequency in the LFP signals recorded during behavior. The across electrode mean and SE are plotted for each of four ferrets.

(J) LFP power distribution. The distribution of power was calculated for the same ferrets passively listening to the same sounds that they discriminated during the behavioural task. We normalized the power spectrum to its peak (which was around 8-10 Hz in both active and passive conditions) and, for each electrode, subtracted the passive from the active values. This plot shows that engaging in the task resulted in relatively more power across a broad range of frequencies from 12 Hz upwards.

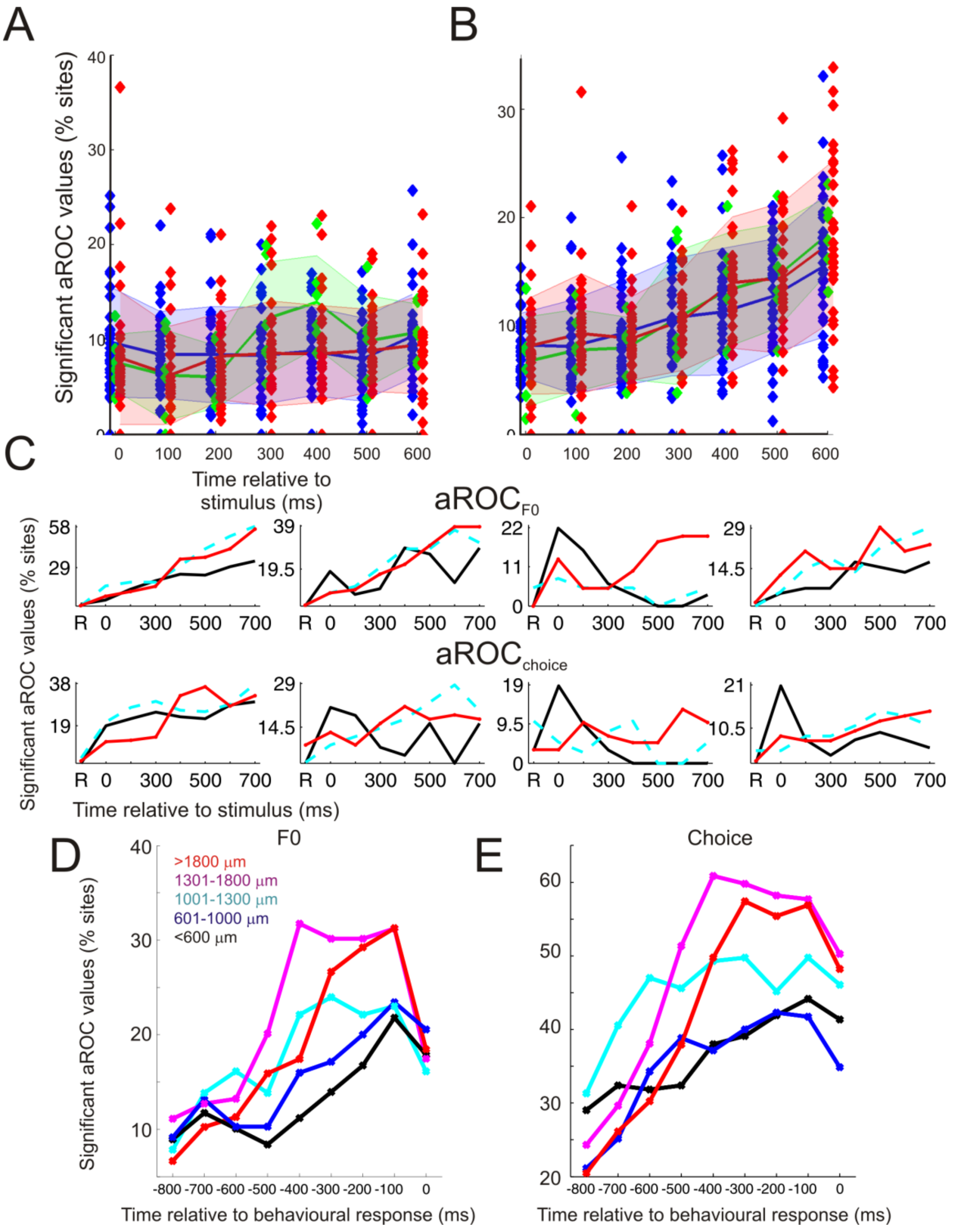

Figure S3, Related to Figure 3. Sensitvitity to F0 and Behavioral Choice across Fields, Depth, and Time
(A and B) the percentage of significant aROC$_{F0}$ and aROC$_{choice}$ values per electrode was calculated for each electrode where there were more than 10 recordings (the mean ±SD per electrode was 76±47.3). This left 34 electrodes in A1/AAF (from four animals), 33 electrodes in the PEG (field PPF and PSF, from four animals), and 6 electrodes (3 from each of two animals) in field ADF and the pseudosylvian sulcal

cortex. There were no significant differences in the proportion of significant recording sites by cortical field (two-way ANOVA analysis time bin and cortical field as factors, $aROC_{F0}$ $F_{(2,560)}=1.11$ $p = 0.32$ $F_{(7,560)}=5.74$, $p<0.001$, interaction $F_{(14,560)}=1.2$ $p=0.27$. $aROC_{choice}$ $F_{(2,560)}=2.49$ $p = 0.08$ $F_{(7,560)}=25.67$, $p<0.001$, interaction $F_{(14,560)}=0.65$ $p=0.82$). We therefore examined whether aROC values that were significantly different from chance differed across cortical fields (using all recordings, 2809 (4), 649 (3), 2270(4) (total sites, (number of animals)) for primary, anterior and posterior fields respectively). The results of this analysis are shown in Figure 2E,F in the main manuscript.

(C) Individual data exploring differences by cortical depth. When considering single animals we divided our data into three depth bins (<1000 μm, 1001-1300 μm and >1300 μm) equally spaced to include the same range as the across-animal comparison in figure 3. For each of the four animals, the percentage of sites yielding a significant $aROC_{F0}$ or $aROC_{choice}$ value is plotted in the top and bottom row respectively.

(D and E) Response timed distribution of significant sites by depth. Data from all four animals are plotted (as in Figure 3C,D) and the same trend for a greater number of significant sites to be found in the deeper cortical layers is evident.

Supplemental Experimental Procedures

Implantation of Recording Electrodes

WARP-16 drives (Neuralynx, Montana, USA) were loaded with tungsten electrodes (FHC, Bowdoin, USA) and prepared for implantation following methods described in [3]. General anesthesia was induced with an intramuscular injection of medetomidine (Domitor; 0.022 mg/kg; Pfizer, Kent, UK) and ketamine (Ketaset; 5 mg/kg; Fort Dodge Animal Health, Kent, UK). Animals were intubated and ventilated, and anesthesia was then maintained with ~1.5% isofluorane in oxygen throughout the surgery. An i.v. line was inserted and a continuous infusion, 5 ml/h, of lactated Ringers solution was infused. Vital signs (body temperature, end-tidal CO_2 and the electrocardiogram) were monitored. The temporal muscles were retracted, and bilateral craniotomies were made over auditory cortex. The WARP-16 drive was implanted over auditory cortex and sealed above the dura with silastic (FHC) using methods adopted from [3]. The array drives were secured by a bone cement pedestal built around and between them. Two securing nuts were placed within this pedestal, which later allowed us to hold the ferret's head still while advancing electrodes into the brain. We also placed two small stainless steel screws in the skull near the array drives to serve as a low-impedance electrical ground (reference) for the electrodes. Some temporal muscle and skin were removed in order to close the remaining muscle and skin smoothly around the edges of the implant. Animals were allowed to recover for a week before the electrodes were advanced into auditory cortex. Pre-operative, peri-operative and post-operative analgesia and anti-inflammatory drugs were provided to animals under veterinary advice.

Insertion of Microelectrodes into Auditory Cortex

The animal was sedated (Domitor; 0.1mg/kg; i.m.) and the head was then stabilized using the implanted nuts. Electrodes were individually advanced in small steps (~20 µm) with impedance measurements made at each position. The cortical surface was determined to be the point at which we observed a sudden drop in impedance, which indicated that the electrode had advanced beyond the silastic surrounding the WARP-16 drive and onto the dura. The first neural signals were usually recorded within a few hundred microns of this depth. Electrodes were moved incrementally, roughly every two weeks, in steps of at least 50 µm (typically 100 µm). At no point did we 'search' for neural activity; rather all electrodes were advanced and recordings were commenced at the new location. Sedation was reversed after moving electrodes (<60 minutes sedation) with atipamezole (Antisedan; 0.5mg/kg,i.m.; Pfizer).

Voltage signals were recorded, amplified up to 20,000 times, and digitized at 25 kHz. Data acquisition was performed using Tucker-Davis Technologies System 3 multichannel recording systems, together with desktop computers running BrainWare software (Tucker-Davis Technologies, Alachua, USA) and custom scripts written in MATLAB (MathWorks Inc., Natick, USA).

Data Processing

To ensure that data were not influenced by motion artifacts, whole sessions were discarded if the across-trial mean RMS value calculated from the last 500 ms of the trial exceeded two standard deviations above the mean RMS calculated for the first 500 ms of each trial. Such sessions were usually instances when there was a poor connection between the headstage amplifier and one or more channels of the electrode array. In addition, individual trials had to pass the following two exclusion criteria. First, any trial in which the mean RMS power calculated across the whole trial exceeded three standard deviations above the across-trial mean was discarded. Second, for each trial, the RMS power was calculated in three 300 ms wide time bins placed: (a) immediately before reference sound onset (when the animal positioned her head within the central "start" port to initiate a trial); (b) over the last 300 ms of the target sound, just prior to target sound offset; and (c) from 400 ms after target sound

offset. If the mean RMS power in either of the later two windows was more than twice that in the earliest window, then that trial was discarded. Catch trials, during which the F0 of the target and the reference sound were the same, and correction trials, during which trials were repeated following an incorrect response, were also excluded from analysis. Any session in which more than 30 trials passed these criteria were included in further analysis, yielding a total of 11,445 recordings (across all sessions and electrodes), corresponding to recordings in 1,120 unique brain locations, made from a total of 90 recording electrodes across the four animals. On average 44 ±22% (mean ±SE) of implanted electrodes yielded signals in any one testing session.

Spiking responses were extracted offline from the broadband voltage signal after filtering from 200-5000 Hz. Spikes were detected and clustered into "units" using algorithms adopted from Wave_Clus [4]. To remove potential motion artifacts from the spike signals, the following measures were used. First, if the filtered voltage signals on a given electrode during a given session were too variable, then this recording was deemed "unstable" and the signal was excluded from further analysis. More specifically, the recording on the electrode was considered unstable if the standard deviation of the median signal power across 1-second recording windows was over 5 times greater than the median signal power overall. Secondly, if the recordings on <8 (of the possible 16) electrodes in one hemisphere were stable (as defined above) in a given recording session, then data recorded from that hemisphere during that session were discarded. Thirdly, any spike that occurred simultaneously on over 5 electrodes was considered a motion artifact and discarded from analysis. Finally, the spike shapes, raster plots and autocorrelation of spikes for each clustered unit were visually inspected, and any units that did not look neural (e.g. if the spike shape was too narrow or oscillatory) were excluded from further analysis.

Only units with spike rates that were driven by the target stimuli (t-test, $p < 0.05$) were included in the ROC analysis. In total, 1010 multi-unit responses were included in the ROC analysis. Spike counts were calculated over 200 ms running windows and aROC values were calculated from spike rates using the algorithm described for the RMS LFP values.

Bootstrapping aROC Significance

Trials were categorized according to whether the target sound was higher or lower in F0 than the reference sound and ROC analysis was employed (as described in Results and Discussion) to determine how well the neural signal (i.e. RMS LFP power) distinguished either the F0 class (high vs. low) of the target sound or the behavioral choice (left vs. right) that the animal made. The area under the resulting ROC curve (aROC) was used as our measure of discrimination performance. We determined whether the aROC value obtained was significantly greater than chance (i.e. an area of 0.5) using a bootstrapping procedure. LFP signals were randomly sampled, with replacement, across trials such that the assignments between the LFP values and the classification (either target F0 or behavioral response) were randomized and aROC values were computed for these datasets. Significance was assessed by comparing the original observed aROC value against the distribution of 1,000 bootstrapped aROC values obtained by such random sampling, which will produce a distribution of aROC values centered around 0.5, which was then reflected about 0.5 in order to limit aROC values to between 0.5 and 1. A significance level of 0.0125 was chosen to take into account two factors; first, that each series of RMS values were input to two ROC analyses, one to determine the $aROC_{F0}$ (i.e. the aROC for discriminating whether the target sound was high F0 or low F0) and one for the $aROC_{choice}$ (i.e. the aROC for discriminating whether the animal chose the left or right response spout), and, second, that our analysis windows were overlapping (200 ms duration, 100 ms overlap). Therefore, an observed $aROC_{F0}$ or $aROC_{choice}$ value was deemed statistically above chance if it exceeded the 98.75^{th} percentile (i.e. 1-(0.05/4)) of the bootstrapped aROC distribution. On average, 36.7% of the LFP signals within any testing

session showed a significant pitch response, and 42.3% of electrodes exhibited significant choice sensitivity (in at least 1 time bin).

Selection of "Best" Sessions
It was not uncommon that several recording sessions were obtained for any one electrode location, but the number of sessions obtained for each recording site varied. We elected to take the best, rather than the average session at each site because several external factors could have negatively influenced the quality of the recordings, but no obvious ones would have boosted neural discrimination of low and high targets. The best session was selected in the following manner. At each location and every time window, we first computed the proportion of sessions in which the calculated aROC value was significantly greater than chance. Where >25% of recorded sessions had a significant aROC value, we simply selected the session with the highest value. When <25% of sessions were classified as significant, we determined whether, for any of those sessions, at least two adjacent time bins had a significant aROC value. Since the time bins were overlapping, statistically significant values are to be expected across consecutive bins. Where more than one session had two adjacent significantly informative time bins, we selected the one with the highest aROC. In cases where no sessions met these requirements, we simply selected the session with the highest aROC value, but classified the value obtained in that session as not significantly different from chance. This procedure was applied to both $aROC_{F0}$ and $aROC_{choice}$ values. We informally observed that the best sessions across a site tended to be those with the highest signal-to-noise ratio or the largest number of trials. Overall, the type of session (i.e. "easy", with just two target F0 values, or "difficult" sessions with 7 target F0 values) did not influence the likelihood of a session being classified as the "best" ("easy" sessions formed 24.0% of the best sessions and 23.2% of all sessions). The behavioral performance in a session also did not influence this outcome (mean behavioral performance for "best" sessions was 76.9% correct, compared to 77.6% correct for all sessions). We report the data for all sessions (rather than the best) in all analyses except the analysis by cortical depth where reporting a single value per recording location was essential to perform a valid comparison.

Analysis of Responses by Cortical Depth
In order to analyze how cortical depth influenced response properties we divided recordings into 5 depth bins containing equal numbers of recordings using the 20th centiles of the cumulative distribution of recordings by depth. These resulted in bins that spanned from 0-600, 601 - 1000, 1001 – 1300, 1301-1800 and >1800 μm.

Assigning Electrodes to Cortical Fields
We used a range of anatomical and physiological measures to determine which cortical fields our electrodes were placed in. At each recording location we estimated characteristic frequency using methods described in [5], using a series of pure tone stimuli presented at multiple frequencies (150 Hz-20 kHz, in $1/5^{th}$ octave bins) and intensities (30-80 dB SPL in 10 dB steps). At the end of the recordings, the animals were overdosed with barbiturate, perfused with paraformaldehyde fixative, and the brain was removed for inspection. The tracks left by the recording electrodes were clearly visible on the cortical surface. Supplemental Figure 1F-K shows their locations relative to the suprasylvian and pseudosylvian sulci. Each site is marked in a color that reflects the multiunit characteristic frequency (averaged across all recordings at all depths within the recording tract). White circles indicate electrodes in which it was not possible to derive an estimate of characteristic frequency as the units recorded did not respond to pure tones. In addition, the fixed brains were sectioned and stained with cresyl violet. Sections were examined under a light microscope to confirm the location of the recording electrodes, and to localize (and exclude from further analysis) a small number of recording sites deemed to have been within the white matter.

For analysis purposes, we divided the electrode penetrations into three groups: those in the primary fields (A1 and AAF, 35 electrodes, 6 cortices in 4 animals), those in the posterior fields (PPF and PSF, 47 electrodes, 6 cortices in four animals) and the remaining electrode penetrations, which were distributed in either field ADF or in the pseudosylvian sulcus (12 electrodes, three cortices in three animals). We did not attempt a more fine-grained assignment of penetrations to cortical fields. Few differences between the responses properties of cells in either A1 and AAF or PPF and PSF have been characterized using either simple [5, 6] or complex sounds [7-9] which could serve as physiological criteria for such an assignment. An accurate assignment into these 6 fields requires dense sampling and tonotopic mapping, which were not possible in this chronic preparation.

Supplemental References

1. Liu, J., and Newsome, W.T. (2005). Correlation between speed perception and neural activity in the middle temporal visual area. J Neurosci *25*, 711-722.
2. Liu, J., and Newsome, W.T. (2006). Local field potential in cortical area MT: stimulus tuning and behavioral correlations. J Neurosci *26*, 7779-7790.
3. Eliades, S.J., and Wang, X. (2008). Chronic multi-electrode neural recording in free-roaming monkeys. J Neurosci Methods *172*, 201-214.
4. Quian Quiroga, R., Nadasdy, Z., and Ben-Shaul, Y. (2004). Unsupervised spike detection and sorting with wavelets and superparamagnetic clustering. Neural Computation *16*, 1661-1687.
5. Bizley, J.K., Nodal, F.R., Nelken, I., and King, A.J. (2005). Functional organization of ferret auditory cortex. Cereb Cortex *15*, 1637-1653.
6. Kowalski, N., Versnel, H., and Shamma, S.A. (1995). Comparison of responses in the anterior and primary auditory fields of the ferret cortex. J Neurophysiol *73*, 1513-1523.
7. Bizley, J.K., Walker, K.M., King, A.J., and Schnupp, J.W. (2010). Neural ensemble codes for stimulus periodicity in auditory cortex. J Neurosci *30*, 5078-5091.
8. Bizley, J.K., Walker, K.M., Silverman, B.W., King, A.J., and Schnupp, J.W. (2009). Interdependent encoding of pitch, timbre, and spatial location in auditory cortex. J Neurosci *29*, 2064-2075.
9. Walker, K.M., Bizley, J.K., King, A.J., and Schnupp, J.W. (2011). Multiplexed and robust representations of sound features in auditory cortex. J Neurosci *31*, 14565-14576.
